# Human Activities Recognition in Android Smartphone Using WSVM-HMM Classifier

**DOI:** 10.1007/978-3-030-51517-1_35

**Published:** 2020-05-31

**Authors:** M’hamed Bilal Abidine, Belkacem Fergani

**Affiliations:** 8 Digital Research Centre of Sfax, Sfax, Tunisia; 9 Institut Mines-Télécom, CNRS, Paris, France; 10 Université de Sherbrooke, Sherbrooke, QC Canada; 11 Digital Research Centre of Sfax, Sfax, Tunisia; 12 University of Sfax, Sfax, Tunisia; Laboratoire d’Ingénierie des Systèmes Intelligents et Communicants, LISIC Lab., Electronics and Computer Sciences Department, University of Science and Technology Houari Boumediene (USTHB), Algiers, Algeria

**Keywords:** Activity recognition, Classification, Weighted SVM, HMM

## Abstract

Being able to recognize human activities is essential for several applications such as health monitoring, fall detection, context-aware mobile applications. In this work, we perform the recognition of the human activity based on the combined Weighted SVM and HMM by taking advantage of the relative strengths of these two classification paradigms. One significant advantage in WSVMs is that, they deal the problem of imbalanced data but his drawback is that, they are inherently static classifiers - they do not implicitly model temporal evolution of data. HMMs have the advantage of being able to handle dynamic data with certain assumptions about stationary and independence. The experiment results on real datasets show that the proposed method possess the better robustness and distinction.

## Introduction

The advancement of technologies has facilitated the monitoring of human activities through the embedded sensors in a smartphone. Recently, smart phones, equipped with a rich set of sensors, are explored as alternative platforms for human activity recognition (HAR) [1, 2]. HAR technology aims at recognizing the behavior and activities of users through a series of observations, which has wide application [3, 4] in different areas, such as healthcare and military monitoring.

With smartphones becoming an integral part of daily human life [5], they are being preferred as the most usable appliances that could recognize human activities due to its powerful in terms of mobility, user-friendly interface, network capability, strong CPU, memory, and battery. They contain a large number of hardware sensors such as accelerometer, gyroscope, temperature, humidity, light sensor, and GPS receiver.

The human sensor based activity recognition is a combination of sensor networks hand-in-hand with the data mining and machine learning techniques [6]. The smartphones provide enormous amount of sensor data for one to understand the daily activity patterns of an individual.

The basic procedure for mobile activity recognition involves i) collection of labelled data, i.e., associated with a specific class or activity from users that perform sample activities to be recognized ii) classification model generation by using collected data to train and test classification algorithms iii) a model deployment stage where the learnt model is transferred to the mobile device for identifying new contiguous portions of sensor data streams that cover various activities of interest. Sensor data can be processed in real-time or logged for offline analysis and evaluation. The model generation is usually performed offline on a server system and later deployed to the phone to recognize the activity performed.

Recently, several authors [7, 8] have proposed many applications related to activity recognition on multiple body positions. Most of the work, like Ahmad [9], Tran [10], Awan [11], Shoaib [12], and Abidine [13], consider a single classifier approach to study activity recognition using smartphones. For the classification, SVMs are popular [8, 14]. It is also the case for HMMs [15] which they commonly used for time-series activity recognition. However, there is very limited number of publications in the literature that investigate the application of the WSVM classifier for smartphone data, and no one is found about applying the latter one on smartphone data or even on HAR system’s datasets. Building a system with high precision to accurately identify these activities is a challenging task.

In this work, we adopted a new method for physical activity recognition using mobile phones that uses labels outputting WSVM in HMM. WSVM investigated the effect of overweighting the minority class on SVM modeling between the performed activities. HMM is a natural solution to address the activity complexity by ― capturing and smoothing information during the transition between two activities (e.g. Walking and Standing). We also used the feature extraction approach that transforms the original high dimensional data to a lower dimensional feature space. The transformation can be linear or nonlinear. In this project, we employed the linear Principal Component Analysis (PCA) [16] to extract the feature vectors.

## The Proposed HAR System by Combining WSVM-HMM Based PCA

### Overview

**Fig. 1.**
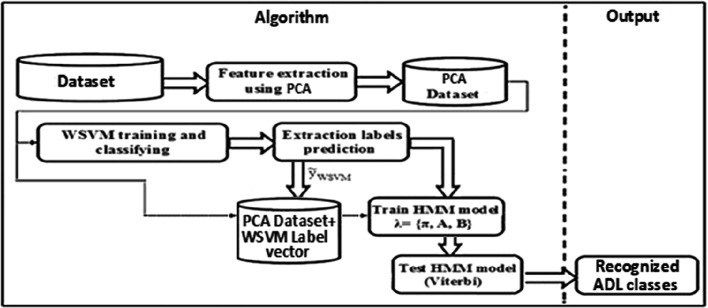
Hybrid WSVM-HMM system based PCA approach.

Figure 1 shows the architecture of the proposed activity recognition system. Among the available labelled data, training and test subsets are chosen using the cross-validation mechanism. The constructed PCA space is then used for training and testing the Weighted SVM classifier. In the second step of the process is a pre-classification by ‘WSVM’, this phase is carried out by the ‘cross-validation’ will generate an estimate of the label vector.

The principal component features concatenated with the WSVM estimated label vector are employed as a new training data to train HMM classifier. The final classification is performed with the ‘Viterbi’ algorithm, by the use of a HMM model.

An estimated label vector is generated by the ‘Viterbi’ algorithm and the system will output the recognized activity (i.e., walking, running, and others).

### Principal Component Analysis (PCA)

PCA [16] is an orthogonal projection-based technique such that the variance of the projected data is maximized. In our case, a large number of features are extracted by prepossessing the raw signals generated from different sensors. It is a widely used technique for dimensionality reduction, feature extraction, and data visualization through the construction of uncorrelated principal components that are a linear combination of the original variables. The PCA components can be counted by performing the eigenvector decomposition of the covariance matrix *S*:

1 $$ S = \sum\limits_{j = 1}^{m} {\left( {(\vec{x})_{j} - \mu } \right)\left( {(\vec{x})_{j} - \mu } \right)^{T} ,\quad \mu } = \frac{1}{m}\sum\limits_{j = m}^{m} {\left( {\vec{x}} \right)_{j} } \,. $$

This problem leads to solve the eigenvalue equation with *λ is the eigenvalue of S and V is the eigenvector corresponding to the λ*:

2 $$ \lambda V = SV,\left| {\left| V \right|} \right| \, = \, 1. $$

Where V = [v_1_, v_2_, …, v_i_], (i = 1, …, n) is the n × n matrix containing n eigenvectors and λ is an n × n diagonal matrix of eigenvalues of the covariance matrix. In Eq. (2), each n dimensional eigenvector v_i_ corresponds to the ith eigenvalue λ_i_.

### Weighted Support Vector Machines (WSVM)

Osuna et al. [17] proposed an extension of the SVM modeling, Weighted SVM algorithm to overcome the imbalance problem by introducing two different penalty parameter $C_{ - }$ and $C_{ + }$ in the primal Lagrangian (Eq. 3) for the minority (*y*_*i*_ = −1) and majority classes (*y*_*i*_ = +1), as follow

3 $$ \begin{aligned} & \mathop {\hbox{min} }\limits_{s,b,\zeta } \frac{1}{2}w \bullet w + C_{ + } \sum\limits_{{i|y_{i} = 1}}^{{m_{ + } }} {\zeta_{i} } + C_{ - } \sum\limits_{{i|y_{i} = - 1}}^{{m_{ - } }} {\zeta_{i} } \\ & \text{subject}\;\text{to}\,\,y_{i} (s \bullet \varPhi (y_{i} ) + b) \ge 1 - \zeta_{i} ,\;\zeta_{i} \ge 0,\;i = 1, \ldots ,m\;. \\ \end{aligned} $$

$m_{ + }$ *(resp.* $m_{ - }$*)* the number of positive (resp. negative) instances in the initial database *(*$m_{ - } + m_{ + } = m$*).* Solving the formulation dual of WSVM [17] gives a decision function for classifying a test point $y \in R^{p}$

4 $$ f(x) = \text{sgn} \left( {\sum\limits_{i = m}^{{m_{sv} }} {\alpha_{i} y_{i} K(x,x_{i} ) + b} } \right)\;. $$

We used the Gaussian kernel as follows: $K(x,y) = \exp \left( { - \left\| {x - y} \right\|^{2} /2\sigma^{2} } \right)$. Some authors [17–19] have proposed adjusting different cost parameters to solve the imbalanced problem. To extend Weighted SVM to the multi-class scenario in order to deal with *N* classes (daily activities), we have shown in [20] that the cost of misclassifying a point from the small class should be heavier than the cost for errors on the large class. They used different misclassification *C*_*i*_ per class, use this conclusion can get a satisfactory result. By taking *C*_−_ = *C*_*i*_ and *C*_+ =_ *C,* with $m_{ + }$ and $m_{i}$ be the number of samples of majority classes and number of samples in the *i*^th^ class, the main ratio cost value ***C***_***i***_ for each activity can be obtained by:

5 $$ C_{i} = \text{round}(C \times \left[ {m_{ + } /m_{i} } \right]),\;i = 1, \ldots ,N. $$

### Hidden Markov Model (HMM)

HMM [21] comprises two parts: Markov chain and stochastic process. Markov chain, whose output is a sequence of state, can be described by the initial probability distribution for the states (π) and the state transition matrix (A), while stochastic process whose output is a sequence of observed values, is described by the observation probability matrix (B). Thus, a HMM can be described as:

6 $$ A = \text{a}_{{\text{ij}}} = P(y_{t} = j|y_{{t\text{ - 1}}} = i)\;\text{and}\;\sum\limits_{j = 1}^{N} {a_{ij} } = \text{1} $$

7 $$ B = \left[ {b_{j} \left( {O_{t} } \right)} \right] $$

8 $$ b_{j} \left( {O_{t} } \right) = \, P\left( {q_{k + 1} = O_{t} / \, q_{t} = i} \right) $$

9 $$ \pi = [\pi_{ 1} ,\pi_{ 2} \ldots ,\pi_{N} ] $$

10 $$ \pi_{\text{i}} = P\left( {q_{0} = i} \right). $$

- With*: i*, *j* ϵ {1,2, …, *N*}
- *O*_*t*_: Vector of observations

A standard HMM is a generative probabilistic model, which generates hidden states *y*_*t*_ from observable data *x*_t_ at each discrete time instant. In our case the hidden variable is the activities that the subject was performing at a given time step and the observable variable is the vector of sensor readings. HMM model mainly works on two basic principles as follows: the observable variable at time *t*, namely *x*_t_, depends *only* on the hidden variable *y*_t_. The hidden variable at time *t*, namely *y*_*t*_, depends *only* on the previous hidden variable *y*_*t*−1_.

Learning the parameters of these parameters corresponds to maximizing the joint probability p(x, y) between the sensor data and activities in the training data. The joint probability therefore factorizes as follows:

11 $$ P(\text{x},\text{y}) = \prod\limits_{t = 1}^{T} {p(y_{t} |y_{t - 1} )} p(x_{t} |y_{t} )\;. $$

The main aim of this model is to determine the best hidden state sequence from the observed output sequence that maximizes *p*(x, y).

## Experimental Results and Analysis

### Datasets

We validate our method on three public datasets whose information is summarized in Table 1. The first dataset used is from [22]: the Human Activity Dataset (HAR). The second dataset (HAPT) [23] with Postural Transitions is similar to previous dataset, further, it includes postural transitions in addition of the previous version of the dataset Records. The third dataset is from [24], titled Wireless Sensor Data Mining (WISDM). All datasets have been recorded by means of Android smartphone. For the annotation of the activities, the video-recorded is used to label the data manually. The HAR and HAPT datasets provide a large extracted features extracted by prepossessing the raw signals generated from sensors.

**Table 1.** Summary of datasets used in the evaluation

Houses	HAR	HAPT	WISDM
Nb of subjects	30	30	29
Annotation	Video	Video	Graphical user interface
F_Sampling_ (Hz)	50	50	20
Features	561	561	6
Smartphone	Samsung Galaxy SII	Samsung Galaxy SII	Cell Phone
Position	Waist	Waist	Front leg pocket
Sensors	Accelerometer and gyroscope	Accelerometer and gyroscope	Accelerometer
Activities	6	12	6

### Results

These algorithms are tested under MATLAB environment and the WSVM algorithm is tested with implementation LibSVM [25] using Gaussian kernel is used for all the datasets. Each training dataset is normalized before classification within a range of [−1, 1]. We optimized the SVM hyper-parameters (*σ, C*) for all training sets in the range [0.1, 0.2, 0.5, 1] and {0.1, 1, 5, 10, 100}, respectively, to maximize the error rate of five fold- cross validation technique. The optimal parameters *σ*_*opt*_ = 0.9, 0.9, and 0.8 are found to be optimal the training dataset of HAR, HAPT, and WISDM, respectively. We show in the Table 2 that the fusion of principal component features with WSVM-HMM makes the model more robust, achieving better performance. One also notices for HAR dataset that the multi-class WSVM method improves the classification results over MC-SVM, MC-HF-SVM and HMM classifiers used alone. On the other hand, the results also show that WSVM outperforms HMM for recognizing activities for all datasets except for the HAPT dataset.

**Table 2.** The micro-averaged measures: Recall, Precision, F-measure and Accuracy for all approaches in (%). Bold values are the results for our approach for each dataset.

Datasets	Approach	Recall	Precision	F-measure	Accuracy
HAR	MC-SVM [8]	89.6	89.9	89.7	89.3
	MC-HF-SVM [8]	89.3	89.2	89.2	89.0
	WSVM	92.4	91.6	91.9	93.9
	HMM	89.2	90.2	89.7	93.7
	**Proposed**	**94.0**	**96.7**	**95.3**	**94.9**
HAPT	WSVM	96.0	92.4	94.1	86.1
	HMM	98.3	97.1	97.7	96.5
	**Proposed**	**97.3**	**99.0**	**98.1**	**96.8**
WISDM	J48 [26]	81.7	–	–	85.1
	LogisticRegression [26]	68.4	–	–	78.1
	MultilayerPerceptron [26]	80.4	–	–	91.7
	WSVM	83.4	76.5	79.8	81.4
	HMM	79.4	80.0	79.7	84.9
	**Proposed**	**91.9**	**79.8**	**85.4**	**92.3**

In terms of reducing the datasets, the feature reduction identifies the most relevant features for the learning process. We notice that PCA features can improve the discrimination between different activities than the original features. For WISDM the performances of activity recognition are low than HAR and HAPT datasets with 561 features. This is explained by the number of features (6) for WISDM is not sufficient when using PCA algorithm. Another reason to the lowest accuracy in WISDM dataset is attributed to the use only the accelerometer sensor comparatively to the HAR and HAPT that use the both accelerometer and gyroscope sensors.

To get a detailed knowledge of the performances on each class corresponding to current activity for the HAR dataset with six different activities. We calculate the confusion matrix of the proposed method in Table 3. From these tables, we see that the best performances were obtained for the proposed method for all classes, in particular for the static activities (Sitting and Standing).

**Table 3.** Confusion matrix of activities for the proposed method on the HAR dataset.

Activities	Walking	W. Upstairs	W. Downstairs	Sitting	Standing	Laying
Walking	**97.1**	2.1	0.7	0.0	0.0	0.1
Walking. Upstairs	1.2	**96.2**	2.4	0.1	0.1	0.0
Walking. Downstairs	0.7	2.2	**97.1**	0.0	0.0	0.0
Sitting	0.6	0.0	0.1	**83.5**	12.2	3.6
Standing	0.1	0.2	0.2	7.4	**91.4**	0.7
Laying	0.0	0.0	0.3	0.8	0.3	**98.6**

In the Table 3, 96.2% of ‘W. Upstairs’ activity instances are correctly recognized, while 2.4% goes into ‘W. Downstairs’ and 1.2% are confused with ‘Walking’ activity. The similar classes such as ‘Walking’, ‘W. Upstairs’, and ‘W. Downstairs’ show similar trend of sharing errors among each other. The reason is the similar status of smartphone when the user does these dynamic activities. We notice that the static activities share errors among each other. 12.2% of ‘Standing’ activity instances are confused with ‘Sitting’ activity and 7.4% of ‘Sitting’ activity instances are confused with ‘Standing’ activity. Intuitively, this can be explained by the fact that the patterns in the acceleration data between these activities are somewhat similar.

## Conclusion and Future Work

Experimental results of the hybrid model presented demonstrate how it can be effectively employed for activity recognition of static and dynamic activities. It obtains a significant performance. Specifically, we show how the hybrid system obtained by using the WSVM label output a new feature added to the reduced data for training and testing HMM outperforms other well known supervised pattern recognition approaches. We consider that WSVM approach has great potential to deal the imbalance class in this human activity recognition problem. However, it must be noticed that hybridizing these schemes implies a more complex system. Fortunately, the training phase in a deployed activity recognizer is usually done offline, so we do not consider such growth of complexity a real problem in our domain.
